# An academic perspective of participation in healthcare redesign

**DOI:** 10.1186/s12961-019-0486-2

**Published:** 2019-11-20

**Authors:** Sarah Jane Prior, Carey Mather, Andrea Miller, Steven Campbell

**Affiliations:** 1 School of Medicine, College of Health and Medicine, University of Tasmania,, Cradle Coast Campus, Mooreville Road, Burnie, Tasmania 7320 Australia; 2 School of Nursing, College of Health and Medicine, University of Tasmania,, Newnham Campus, Launceston, Tasmania 7250 Australia

**Keywords:** Healthcare redesign, academic, accountability, partnerships, integrity

## Abstract

Healthcare redesign, based on building collaborative capacity between academic and clinical partners, should create a method to facilitate flow between the key elements of health service improvement. However, utilising the skills and resources of an organisation outside of the health facility may not always have the desired effect. Accountability and mutually respectful relationships are fundamental for collaborative, sustainable and successful completion of clinical research projects. This paper provides an academic perspective of both the benefits of academic involvement in facilitating healthcare redesign processes as well as the potential pitfalls of involving external partner institutions in internal healthcare redesign projects.

## Plain English summary

Health service delivery improvement can be a complicated task requiring much time and resources. Re-designing a service that is problematic requires clinical knowledge and skills in that area. However, this process also requires knowledge and skills to be able to assess what is not working, and why, and to develop solutions to improve the services through a variety of ways. Often, hospitals or other healthcare providers request assistance from academic staff from universities to ensure that these tasks are completed, and that minimal time is taken from healthcare professionals and their patients. Historically, relationships between university academics and healthcare providers have faced several challenges. This paper discusses the challenges, and benefits, from the academics’ point of view.

## Background

Across Australia and internationally, healthcare services are faced with challenges associated with a rising demand for medical care. The delivery of efficient, best-practice care is increasingly becoming the result of the redesign of healthcare services, a time-consuming and resource-rich process. External facilitation of healthcare redesign initiatives provides a means of bringing together key stakeholders to achieve the intended outcomes by committing resources at many different levels. In Australia, healthcare has traditionally partnered with academic institutions through various roles, including education, recruitment and community projects [1]. Collaborative healthcare redesign has also been utilised globally to improve patient care and deliver best practice health services [2–5].

Recent problems in variations in the quality of healthcare in Australia has led to a shift in the direction of healthcare redesign rather than solving problems as they occur. This method of quality improvement has been highlighted by the number of Australian projects, including the redesign of healthcare services at Flinders Medical Centre in South Australia [6], where patient flow was improved in the overcrowded emergency department. Additionally, the development of local postgraduate, work-integrated courses specifically designed to deliver healthcare redesign projects [7] have been developed. For example, the University of Tasmania delivered several health service redesign projects throughout Tasmania to improve the patient healthcare journey, including improving the efficiency of the surgical theatre [8] and the reduction of outpatient waitlists in the north of the state [9].

Healthcare redesign is a phasic process often performed as a single service or discipline in the interests of time and resources within the healthcare system itself. Financial restrictions often mean that extra staff are unable to be employed specifically to focus on redesign initiatives, potentially leading to conflicts in workload such as prioritisation of tasks and time management. The employment of academic institutions to provide support throughout healthcare redesign can create more capacity for research and evidence-based service development as well as providing extra resources for the healthcare facility to deliver improved health services. Academics not only provide human resources to deliver the clinical expectations from healthcare redesign, they also offer professional outputs such as ethical approval processes, publication in reputable peer-reviewed health and medical journals, and opportunities for further research and funding to develop clinical practice. The committed resources, including human and fiscal, are necessary tools for sustaining partnerships [10] and will greatly improve the prospect of success in healthcare service redesign.

### What is healthcare redesign?

Healthcare redesign has been broadly defined as “*thinking through from scratch the best process to achieve speedy and effective care from a patient perspective, identifying where delays, unnecessary steps or potential for error are built into the process, and then redesigning the process to remove them and dramatically improve the quality of care*” [11]. As a strategy for implementing quality improvements and initiatives and to deliver evidence-based, best practice, patient-centred care, the process of healthcare, or clinical, redesign is an important tool for healthcare reform. This process of improving the quality of current health services and systems generally occurs in five main phases (outlined in Table 1), as described previously [6].

**Table 1:** Overview of healthcare redesign phases [6]

Phase		Overview
1	Mapping	Detailed view of the existing processes, includes role delineation, responsibilities and what is happening as a patient moves through a healthcare service
2	Diagnosis	All the information from the mapping is pulled together to determine where problems exist and why; a step-by-step questioning process opens up the current systems and allows processes to be defined and issues revealed
3	Solutions	Involves more clinical input, although a research component is necessary to ensure evidence-based solutions; during this phase, the opinions of all stakeholders is required to develop ways of moving forward and of improving the current systems as diagnosed in the first phase of redesign
4	Implementation	The most difficult step – implementing the novel solutions previously identified; involves combining project management skills with managing the human dimension of change in a complex healthcare system
5	Evaluation	Following implementation of solutions, an evaluation process must be put in place to measure the redesign outcomes; this may be patient outcomes, organisational outcomes or other quality and safety outcomes

Healthcare redesign follows the patient journey and identifies areas most in need of change to deliver improved healthcare services. Redesign strategies can be applied to any facet of healthcare and have been shown to be successful in several quality improvement initiatives. For example, Choe et al. [12] reported the success of a clinical redesign methodology for diabetic patients, 69% of whom were able to meet their blood pressure target following a new reminder and self-management system. The Australian national maternity reform agenda utilised a major clinical redesign to establish a midwifery group practice at a tertiary referral hospital in Sydney [13]. This redesign project included the introduction of caseload midwifery care under randomised trial conditions to ultimately improve patient access to midwifery services.

The redesign process requires input from a range of key stakeholders and information from a variety of sources, brought together on a regular basis to initiate and maintain system changes. One of the major steps, and often a significant hurdle in the redesign process, is the engagement of key stakeholders, which may include clinicians, leadership groups, other staff, and consumers or patients. Visible involvement of senior management or leadership groups is essential for driving any change process [14] and enabling a sense of trust and commitment. Patients, carers, families and other consumers also play a major role in defining problems and developing solutions within healthcare redesign. The inclusion of the consumer voice means that patients’ needs are better met and their experience will be improved, which has been shown to lead to improved patient outcomes [15, 16]. Another frequently occurring issue is the high level of time involved in assembling and maintaining the stakeholder group, one of many redesign processes that requires a dedicated role by one team member.

Healthcare redesign can be internally or externally driven, and an appropriate expertise and skill mix is fundamental for the success or failure of implementation in each of the project phases. This expertise includes not only clinical but also administrative, research-focused and patient-centred project skills. Leadership within redesign processes requires expertise, credibility and experience to understand the complexity of the clinical interface and the academic requirements, without compromising the validity of the process.

### Developing partnerships

There are a range of contributing levels within clinical redesign. Any proposed redesign processes need to be cognisant of individuals, organisations and systems [17] that may be impacted and need to be addressed if partnerships are to become successful in obtaining and maintaining project cohesion [18]. Human factors, such as shared vision [19], readiness [20], leadership, assigned workload [19], engagement and ‘buy-in’ [21], ritual or resistance [22], contribute to the successful development or failure of partnerships. Power inequity is a challenge that needs to be acknowledged early in any team development [18]. Sound communication is paramount to promote compliance of implementation plans among stakeholders [19]. Similarly, the influences of the human and physical environment and equipment requirements should not be underestimated. For example, at a systems level, co-governance [23] is important to ensure mutuality of understanding among academic and industry stakeholders. Stewardship of various aspects of the project may vary during the redesign process, depending on the stage and level of guidance required.

The potential for cultural disruption at an organisation level [24] also needs to be considered to ensure that academic and industry partnerships remain interactive, interrelated and dynamic [19]. At an individual level, engagement rather than resistance needs to be promoted to minimise workforce stress arising from the drive towards efficient delivery of healthcare. Parameters around which stakeholders are fundholders and how resources will be distributed require transparent negotiation, including appropriate documentation of meetings, contracts or variations to proposed plans [25].

Levels of partnership may also vary based on where in the clinical redesign pathway participants are enabled or ready to participate [20]. For example, academic involvement can ensure timely, structured development of the diagnostic process and guide the solutions phase when issues are divulged during mapping and diagnosis. The implementation phase needs to be championed by stakeholders involved in the process and those most likely to benefit from the outcomes [26]. The evaluation phase could be managed by members of the academic institution with input from the clinical team [26]. Regardless of the level of partnership is the need to foster rapport development, recognise each stakeholder’s unique contribution, and cultivate trust and mutual respect [1]. Without acknowledgement of each stakeholder’s perspective and expertise, it is likely that the partnership will fail.

### Determining roles in redesign

Within the process of clinical redesign, there are several key roles that need to be established at the outset of the redesign project. Academic involvement instantly relieves pressure from the healthcare organisations to deliver in all these roles. The project steering committee will generally consist of a mix of clinical and managerial staff at varying levels, therein creating a potential issue regarding ownership of the project. The academic is generally employed by the steering committee to function as the project lead. This role includes ensuring that the processes and procedures for clinical redesign are adhered to within the governance framework of research ethics and the healthcare organisation. When healthcare redesign utilises the knowledge and skills of stakeholders from academic institutions and industry, potential is created for collaboration and positive change. However, different levels of partnership may emerge based on the nature and scope of redesign desired. These stakeholders or team members will be responsible for initial mapping, maintaining initiatives and documenting the phases of the project. Agreement about which stakeholders will be responsible for administration of the overall project and how this will be undertaken will need to be clear early in the project, prior to, or in the very early stages of the mapping phase. Often, the administration of projects will be determined by the funding source. If this is the case, it is the responsibility of the project team to nominate stakeholders or team members who will be responsible for ensuring appropriate documentation and milestones are reported to funding bodies in a timely manner. This highlights the requirement for time-rich project team members and ongoing resources, of which healthcare organisations generally have neither.

It is claimed that, for clinical redesign to be successful, all stages of the process need to be clearly visible to all involved [6]. If this process, or any part of the process, is solely undertaken by academics, clinicians may view the process as imposed rhetoric rather than fact [6]. Academics are not employed to manage a patient case load and their input may be viewed as unwanted as there may be a perceived disconnection of academics to the reality of everyday clinical practice. This gap between what is viewed as the best practice within academia and the day-to-day reality of healthcare practice can be a source of friction. Academics are sometimes viewed by those in clinical practice as overly idealist in their approach and are only able to view the world from their ‘ivory towers’ [27]. This is not a new debate, Waring and Bishop [22] contend healthcare redesign has faced similar issues between the competing ‘logic’ gap of managers and healthcare professionals. The ‘logic’ gap may also be viewed in terms of a competing agenda between the two organisations. Sahs et al. [21] contends clinicians may mistrust the academics’ intentions and goals. Clinicians may feel that academics do not have the patient at the centre of the redesign process – rather they are involved as a career development opportunity or to seek funding opportunities [21].

Language can also be a barrier to academic input in healthcare redesign and academics must be aware of the impact of this when communicating with clinical staff and other members of the redesign team. If academic staff present as distant from the clinical practice environment, then trust and respect will be difficult to develop and maintain. The partnership must share a common language as it is important to overcome differing workplace cultures and values [28]. Academics involved in clinical redesign must adapt their language to ensure that the ultimate goals of the redesign are meaningful and relevant to the intended context.

The main aim of clinical redesign is to improve patient experience and care; however, clinicians and managers often have different views on strategies for delivering these goals. The Partnership model [29] depicts two aspects of clinical care – managerial and clinical, which might also be termed evidence-based care. Both management and clinicians can be described as interested in patients; however, there is suspicion from clinicians that the intentions of management are at clinicians’ expense and certainly not for the benefit of patients. From the management perspective, clinicians may be viewed as self-interested and, in some cases, unable to understand the ‘big picture’ of financial pressures and fighting for their own interests. Roles such as those of medical directors are intended to bridge management and clinical hierarchies, with some hybrid roles being highly successful provided that they are not seen to overly support management and, therefore, not perceived by clinicians as losing clinical credibility.

Academics can play a very useful role in terms of bridging the roles and functions of the management and clinical streams. In terms of sustainability, the two streams and the way they work together are important. If management invite the academic institution into a project, then there may be some mistrust by clinicians at the outset. University colleagues need to work hard to assert their independence in the process. Creating balance between management and clinical agendas is more likely to be sustainable than if only one stakeholder group invites the university academics to participate. The development of balance recognises the needs of both the streams of practice and university group members who undertake valuable roles and functions within redesign projects. The lack of acknowledgement of either group by stakeholders is related to the notion of ownership of the project. If management leads the project, then it is less likely to be owned by clinical practice, and therefore less likely to be a sustainable project. For instance, performance data in a project could be produced by management or the university. However, the issue here will be that performance data are about the performance of the clinical team; therefore, the development of the criteria for the collection, the collection itself and the analysis of the data being carried out by the clinicians is a situation which is much more likely to embed change in the project and long after it is supposedly finished given that clinicians own the process. Of course, assistance by academics can be offered in terms of literature review work, which then allows the clinicians to choose the criteria based on a synthesis of previous studies. Additionally, the development of data collection instruments, which make the process easier and more focused, could be performed by academics to support the process of redesign. These examples of academic support for the project rely on ownership by the clinicians, which in most cases translates into sustainability of the project outcomes. Once foundation processes of team building and development of a collaborative approach are complete, the work of planning the redesign project can begin [26].

### Project deliverables/outcomes 

Excluding academic involvement in clinical redesign projects can lead to various external failings, including the lack of quality publications and the ultimate failure to share both the process and results with a wider audience [30]. Dissemination of successful healthcare redesign initiatives is imperative to move forward both the redesign agenda and healthcare systems [31]. Building a base for translating redesign knowledge into clinical practice is stronger with input from academics, who generally have a better knowledge of tools such as ethics processes, publication avenues and external funding opportunities than clinical staff. Having academic involvement in these aspects assists in building a research culture and developing research networks within the clinical practice environment [32]. Furthermore, publication and other dissemination activities, such as conference presentations, reports and sharing positive results via social media, are also ways to celebrate achievement and develop the positive culture required for the longevity and sustainability of the redesign [33]. Caution must be taken by academics to ensure the dissemination of any process or results explicitly includes the partner stakeholders to avoid being seen to take ownership and credit for the process [34].

Lack of ethical approval contravenes academic integrity and has the potential to expose organisations to litigation [25]. Perceived omission of human rights through contravention of the ethical approval framework will terminate a project [25, 30]; therefore, it is imperative to engage the correct mix of stakeholders to ensure ethical approval is granted [25]. Appropriate design to ensure unbiased implementation can minimise poorly designed studies. For example, Rathmell and Sandberg [30] report that projects often grow from a desire to effect change; however, analytical flaws and lack of ethical approval render the work unpublishable [30]. Avoidance of this scenario can occur if academic input is sought prior to the design of any clinical project.

## Conclusion

Academic practice partnerships are undoubtedly fruitful. Too often, questionable clinical practices continue that remain unchallenged. Clinical practice has many practical challenges and academics with clinically credible research skills have their place in solving these problems. Working relationships and ownership of the project needs to be transparent; however, for sustainability, the engagement of clinical staff is paramount. For clinical redesign to become synergistic and successful requires mutual understanding of both the knowledge and skills of academics and clinicians.

## Data Availability

Data sharing is not applicable to this article as no data sets were generated or analysed during the current study.
